# Effectiveness of Medical Management in Heavy Menstrual Bleeding: A Prospective Cohort Study

**DOI:** 10.7759/cureus.101162

**Published:** 2026-01-09

**Authors:** Vasudha Vani Lanka, Medha Davile, Avantika Gupta, Deepika Yadav, Shuchita Mundle

**Affiliations:** 1 Department of Obstetrics and Gynaecology, All India Institute of Medical Sciences, Bibinagar, Bibinagar, IND; 2 Department of Obstetrics and Gynaecology, All India Institute of Medical Sciences, Nagpur, Nagpur, IND; 3 Department of Obstetrics and Gynaecology, All India Institute of Medical Sciences, Bhopal, Bhopal, IND

**Keywords:** abnormal uterine bleeding (aub), combined oral contraceptive pills (cocp), depot medroxyprogesterone acetate, effectiveness, heavy menstrual bleeding (hmb), levonorgestrel intrauterine system (lng-ius), medical management, pbac, pictorial blood assessment chart, tranexamic acid

## Abstract

Introduction: Heavy menstrual bleeding (HMB) is a prevalent gynecological condition among women in the reproductive and perimenopausal age groups, significantly impairing quality of life. While several medical treatment options are available, their comparative effectiveness and patient adherence vary. This study aimed to evaluate the effectiveness of medical management in HMB.

Methods: This prospective cohort study was carried out at a tertiary care institute in Central India from February 2023 to July 2024. The study included 240 women between 13 and 50 years of age presenting with HMB who opted for medical management. HMB was defined by a Pictorial Blood Loss Assessment Chart (PBAC) score of > 100. Women with HMB secondary to pregnancy complications, women needing surgical management, and women with postmenopausal bleeding were excluded from the study. Medical management was selected based on patient preference and clinical suitability. Outcome parameters like change in PBAC score and hemoglobin level were noted at baseline and reassessed at three and six months.

Results: The mean age of participants was 41.03 ± 7.23 years. Baseline median PBAC scores (IQR) were: levonorgestrel intrauterine system (LNG-IUS) 475 (360-690), tranexamic acid 400 (255-600), norethisterone 645 (350-775), injectable depot medroxyprogesterone acetate (inj DMPA) 690 (355-956), and combined oral contraceptive pills (COCP) 380 (259-580). All five groups showed a significant reduction in PBAC scores at three and six months (p < 0.001). The greatest reduction at three months was seen with inj DMPA (78%), while LNG-IUS showed the highest reduction at six months (93%). A statistically significant improvement in hemoglobin levels was also observed over six months in all groups.

Conclusion: Medical management of HMB was effective in reducing menstrual blood loss and improving hemoglobin levels. LNG-IUS offered the most significant benefit with high compliance, but treatment choice should be individualized based on patient needs and clinical profile.

## Introduction

Normal menstrual bleeding is the result of a finely coordinated interplay of endocrine, paracrine, immunological, and hemostatic factors acting on the endometrium. In the late secretory phase, estrogen promotes the upregulation of estrogen (ER), progesterone (PR), and androgen receptors (AR), supporting cellular proliferation and subsequent restoration of the endometrium. Just before menstruation begins, an inflammatory cascade is triggered within the stratum functionalis, characterized by increased pro-inflammatory cytokines and activation of progenitor stem cells originating from the stratum basalis.

During menstruation, elevated levels of prostaglandins such as PGE₂ and PGF₂α enhance contractility of the subendometrial myometrium, aiding in the expulsion of the shedding endometrium. Following menstrual shedding, estrogen plays a key role in the rapid repair and regeneration of the endometrial lining. Angiogenesis induced by vascular endothelial growth factor produced from intravascular neutrophils is also thought to occur in the endometrium during proliferative and secretory phases. Abnormal or incomplete angiogenesis, resulting in abnormal blood vessels with fragile vessel walls, may cause HMB, and several studies have suggested that abnormal endometrial vessel number, structure, or function may play a role in HMB. Aberrations in any of these processes may result in HMB, and medical therapies are directed at correcting one or more of these underlying mechanisms.

Heavy menstrual bleeding (HMB) is a prevalent gynecological condition that can significantly impair a woman’s physical, emotional, social, and economic quality of life. Clinically, it is defined not only by the volume of menstrual blood loss, traditionally estimated as more than 80 mL per cycle, but also by its adverse impact on daily functioning and well-being [1,2]. HMB affects up to 30% of women at some point in their reproductive years and is a leading cause of gynecological consultations and interventions [3]. The diminished quality of life experienced by women with HMB is also a result of the clinical effects of prolonged and excessive blood loss [4].

Management options for HMB include both medical and surgical approaches. Minimally invasive alternatives, such as endometrial ablation and uterine artery embolization (UAE), are also recommended for selected women who have completed childbearing; these procedures effectively reduce menstrual blood loss and improve quality of life, though they carry risks of treatment failure and the potential need for subsequent surgery. While hysterectomy offers definitive treatment, it carries inherent surgical risks (approximately 1%), including injury to adjacent organs, hemorrhage, infection, and anesthesia-related complications, and is therefore typically reserved for cases unresponsive to medical therapy or with coexisting pathology such as fibroids or adenomyosis [5, 6]. Current guidelines recommend medical management as the first-line approach in most women, given its safety, efficacy, and noninvasive nature [7].

Medical therapy for HMB includes hormonal and non-hormonal agents. According to the 2018 National Institute for Health and Care Excellence (NICE) guidelines, medical management can be initiated based on clinical assessment and basic imaging, without extensive investigations [8]. Among the available options, the levonorgestrel-releasing intrauterine system (LNG-IUS) has shown the highest efficacy, followed by combined oral contraceptives and progestins. However, Indian literature on the extent of menstrual blood loss reduction with various medical therapies remains limited. Therefore, this study was conducted to evaluate the effectiveness of medical management in reducing menstrual blood loss, as measured by the Pictorial Blood Loss Assessment Chart (PBAC) [9], at three and six months.

## Materials and methods

This prospective cohort study was conducted in a tertiary care institute in Central India over a period of 18 months, from February 2023 to July 2024. The study protocol was approved by the Institutional Research Cell, followed by the Institutional Ethics Committee of All India Institute of Medical Sciences, Nagpur, India (approval number: IEC/Pharmac/2023/544). 

Study population

Women attending the gynecology outpatient department with complaints of HMB were screened for eligibility. HMB was quantified using the PBAC, where a PBAC score >100 was considered diagnostic of HMB. The PBAC score was calculated based on the degree of saturation of sanitary products and the presence and size of clots (Appendix A) [9].

Inclusion criteria

Women aged 13-50 years presenting with HMB (PBAC score >100) and willing to provide informed consent were included in the study. 

Exclusion criteria

Patients requiring surgical management for HMB (e.g., fibroids with uterine size >12 weeks), those with HMB secondary to pregnancy-related complications, those aged <13 years or >50 years, and those with postmenopausal bleeding were excluded.

After obtaining written informed consent from eligible participants (or guardians of girls under 18 years of age), a detailed medical history was recorded, and a thorough clinical examination was performed. This included demographic details (age, marital status, parity), menstrual history (pattern, duration, frequency, duration of symptoms), and past medical and treatment history.

Physical examination included assessment of pallor, height, weight, body mass index (BMI), abdominal and bimanual pelvic examination, as indicated. A transabdominal or transvaginal pelvic ultrasound was performed to identify structural causes and measure endometrial thickness. Baseline investigations included complete blood count (CBC), blood group, liver function tests (LFT), renal function tests (RFT), thyroid function tests (TFT), coagulation profile (if clinically indicated), and Pap smear in eligible participants. Endometrial sampling was performed using Pipelle in women aged >40 years and in women <40 years with obesity or suspected chronic anovulation.

Participants were educated in their local language on how to maintain the PBAC chart. They were informed about standard medical management options, including tranexamic acid, non-steroidal anti-inflammatory drugs (e.g., mefenamic acid), combined oral contraceptive pills (COCP), LNG-IUS, tablet norethisterone, injectable depot medroxyprogesterone acetate (inj DMPA), and tablet medroxyprogesterone acetate (tab MPA). Treatment regimens were selected based on patient preference and clinical suitability and initiated in standard doses. Participants were followed monthly for the first three months and at six months thereafter. At each follow-up visit, the PBAC score was reassessed to monitor bleeding severity.

Outcome measures

At three and six months, the following outcomes were recorded: effectiveness of treatment was defined as a ≥25% reduction in PBAC score from baseline at three months; hemoglobin level; and compliance with treatment. For oral medication, it was defined as taking the medication as prescribed (yes/no); for LNG-IUS, non-compliance was defined as removal of the device within six months; for inj DMPA, compliance was assumed for three months following administration.

If treatment was discontinued within the first three months, reasons were documented (e.g., inconvenience, adverse effects, lack of relief, unwillingness for device insertion). Participants who did not achieve the target PBAC score reduction (<25%) were evaluated for surgical options based on clinical findings. These patients were excluded from further follow-up of the initial regimen, and those opting for surgery were excluded from continued follow-up; their clinical data were recorded. 

Sample size calculation

Based on outpatient department (OPD) attendance, an estimated eight to 10 patients with HMB presented daily. Of these, approximately two to three patients met eligibility criteria for medical management. Assuming 20 OPD days per month on average and an attrition rate of 50%, the calculated sample size was 240 patients over a 12-month recruitment period. The subsequent six months were a follow-up period.

Statistical analysis 

Data were collected using a pre-designed proforma and entered into Microsoft Excel (Microsoft Corporation, Redmond, WA, USA). Following data cleaning, analysis was performed using IBM SPSS Statistics software, version 25 (IBM Corp., Armonk, NY, USA). Categorical variables like age groups, BMI category, marital status, parity, treatment given, etc., were presented as frequencies and percentages, while continuous variables such as age and BMI were expressed as mean ± standard deviation. Continuous variables like PBAC score and hemoglobin level were assessed for normality using the Shapiro-Wilk test and were found to be non-normally distributed; hence, they were reported as median with IQR. Changes in these variables from baseline to three and six months were analyzed using the Friedman test. A p-value of <0.05 was considered statistically significant for all comparisons. 

## Results

A total of 240 women who fulfilled the eligibility criteria were enrolled in the study. The mean age of participants was 41.03 years (SD ±7.23, 95% CI: 40.11-41.95). The majority of participants (101, 42%) belonged to the 41-45 years age group, while the 21-25 years age group had the fewest participants (six, 2.5%). The mean BMI was 25.8 ± 3.67 kg/m². Over half of the participants (127, 52.9%) were classified as overweight, while only three (1.25%) participants were underweight. Most participants (226, 94.1%) were married, and 13 (5.4%) were unmarried. Regarding parity, 22 (9.1%) participants were nulliparous, 18 (7.5%) had at least one child, and the majority (155, 64.5%) were para 2, followed by para 3 (37; 15.4%) and para 4 (7; 2.9%). The most common presenting complaint was heavy menstrual bleeding with prolonged menses in 128 (53.3%) participants, while heavy and infrequent cycles were the least common complaint in five participants (2.08%). Regarding menstrual cycle pattern, 147 (61.2%) participants reported regular cycles, while 93 (38.7%) had irregular cycles (Table 1). 

**Table 1:** Demographic characters of the participants (n=240) The data are represented as number (n) and percentages (%).

S. No.	Demographic variable	Number of participants n (%)
1	Age, in years	
	13-20	8 (3.3%)
	21-25	6 (2.5%)
	26-30	7 (2.9%)
	31-35	14 (5.8%)
	36-40	38 (15.8%)
	41-45	101 (42%)
	46-50	66 (27.5%)
2	BMI of the participants (kg/m^2^)	
	<18.5 (Underweight)	3 (1.25%)
	18.5-24.9 (Normal)	85 (35.4%)
	25.0- 29.9 (Overweight)	127 (52.9%)
	30.0-34.9 (Obese class 1)	21 (8.7%)
	35.0-39.9 (Obese class 2)	4 (1.6%)
	>40 (Obese class 3)	0 (0%)
3	Marital status	
	Married	226 (94.1%)
	Unmarried	13 (5.4%)
	Widow	1 (0.4%)
4.	Parity	
	Nullipara	22 (9.1%)
	Para 1	18 (7.5%)
	Para 2	155 (64.5%)
	Para 3	37 (15.4%)
	Para 4	7 (2.9%)
	Para >4	1 (0.4%)

Associated comorbidities were observed in several participants, with hypothyroidism being the most common, present in 28 (11.6%) cases, followed by diabetes mellitus in 25 (10.4%) cases and hypertension in 21 (8.7%) cases. Other reported conditions included asthma, ischemic heart disease, sickle cell disease, genital tuberculosis, hyperthyroidism, and dilated cardiomyopathy.

Out of 240 participants, 131 (54.5%) had a structural cause of abnormal uterine bleeding (AUB), while the remaining 109 (45.4%) had non-structural causes. Among the structural causes, adenomyosis (AUB-A) was the most prevalent, followed by endometrial polyp (AUB-P). In the non-structural category, ovulatory dysfunction (AUB-O) was the most common cause, followed by endometrial causes (AUB-E). Histopathological examination of the endometrium revealed a proliferative pattern in 77 (35.3%) participants, secretory endometrium in 53 (24.3%), endometrial polyps in 31 (14.2%), and endometrial breakdown in 25 (11.4%) participants. Other patterns included exogenous hormonal effect (16, 7.3%), fragmented endometrial glands (10, 4.5%), endometrial hyperplasia (4, 1.8%), and atrophic endometrium (2, 0.9%) among 218 participants. Endometrial sampling was not done in 22 (9.1%) participants, as they were all below 35 years of age and without any high-risk factors (Table 2). 

**Table 2:** Distribution of participants based on the PALM-COIEN classification (A) and treatment given (B; n = 240) Treatment given is represented as the number of participants (n) and percentage (%). PALM COIEN: Polyp–Adenomyosis–Leiomyoma–Malignancy / Coagulopathy–Ovulatory dysfunction–Endometrial–Iatrogenic–Not otherwise classified; AUB: abnormal uterine bleeding; LNG-IUS: levonorgestrel-releasing intrauterine system; DMPA: depot medroxyprogesterone acetate; COCPs: combined oral contraceptive pills

Sr No	Variable	Number of participants n (%)
A	PALM-COEINS Classification	
1	AUB-P	30 (12.5 %)
2	AUB-A	55 (22.9%)
3	AUB-L	37 (15.4%)
4	AUB-M	9 (3.7%)
5	AUB-C	4 (1.6%)
6	AUB-O	79 (32.9%)
7	AUB-E	24 (10%)
8	AUB-N	2 (0.8%)
B	Treatment given	
1	LNG-IUS	85 (35.4%)
2	Tranexamic acid	68 (28.3%)
3	Injection DMPA	20 (8.3%)
4	Tab norethisterone	42 (17.5%)
5	COCPs	18 (7.5%)
6	Tranexamic acid + Mefenamic acid	3 (1.2%)
7	Oral medroxyprogesterone acetate	2 (0.8%)
8	Mefenamic acid	2 (0.8%)

The most commonly administered treatment was LNG-IUS in 85 (35.4%) participants, followed by tranexamic acid in 68 (28.3%), tablet norethisterone in 42 (17.5%), inj DMPA in 20 (8.3%), and combined oral contraceptive pills (COCPs) in 18 (7.5%) participants. While the least used therapies were mefenamic acid and oral medroxyprogesterone acetate, opted for by two (0.8%) participants each (Table 2). 

A total of 240 participants who met the eligibility criteria were enrolled in the study and received treatment based on the underlying cause and their individual preferences. Three participants were lost to follow-up, and 15 underwent hysterectomy. Fifteen patients who underwent hysterectomy had persistent heavy menstrual bleeding on prescribed treatment. Nine cases were of adenomyosis, and six had small leiomyomas. Consequently, 18 participants were excluded from the final analysis. 

Among 222 (92.5%) participants who completed the treatment, 189 (85.1%) achieved normal menstrual flow, 33 (14.8%) experienced scanty menstruation, and three participants (1.3%) developed amenorrhea. Among those with amenorrhea, two were using the LNG-IUS, and one was receiving inj DMPA.

Baseline median PBAC scores (IQR) for various treatment groups were LNG-IUS: 475 (360-690), tranexamic acid: 400 (255-600), tab norethisterone: 645 (350-775), inj DMPA: 690 (355-956), COCP: 380 (259-580), the combination of tranexamic acid and mefenamic acid: 590 (550-590), and tab MPA: 347 (280-347). Reduction in PBAC score at six months was statistically significant (p < 0.05) in five treatment groups: LNG-IUS, tranexamic acid, norethisterone, inj DMPA, and COCPs (Table 3). 

**Table 3:** Trend of PBAC score and hemoglobin level with each treatment protocol (n=222) The PBAC score is represented as the median PBAC score, and the hemoglobin level is represented as the median hemoglobin level *The level of significance P <0.05 (p value was calculated by using related sample analysis, Friedman’s two-way analysis of variance by Ranks for both PBAC score and hemoglobin level) PBAC: Pictorial Blood Loss Assessment Chart; LNG-IUS: levonorgestrel-releasing intrauterine system; DMPA: depot medroxyprogesterone acetate; COCPs: combined oral contraceptive pills

Treatment given (n=222)	Trend of PBAC score with each treatment protocol				Trend of hemoglobin level with each treatment protocol			
	Median (IQR) PBAC score at baseline	Median (IQR) PBAC score at 3 months	Median (IQR) PBAC at score 6 months	P-value	Median (IQR) hemoglobin at baseline	Median (IQR) hemoglobin at 3 months	Median (IQR) hemoglobin at 6 months	P-value *
LNG-IUS (n=85)	475(360,690)	120(62,175)	32(6,70)	<0.001	10.2 (9-11.3)	10.9 (10-12)	11.4 (10.6-12.2)	<0.001
Tranexamic acid (n=68)	400(255,600)	150(100,200)	60(50,102)	<0.001	10.6 (9.3-11.5)	11 (9.6-12)	11.5 (10.9-12.5)	<0.001
Injection DMPA (n=20)	690(355,956)	150(53.5,375)	60(14.5,150)	<0.001	10.3 (6.8-11.6)	11 (9.4-12.7)	11.2 (10.6-12.6)	0.022
Tab norethisterone (n=42)	645(350,775)	265(180,522)	150(60,220)	<0.001	10 (8.1-11.5)	11.5 (10.3-12.3)	11.4 (10.7-12.5)	<0.001
COCP (n=18)	380(259,580)	180(120,330)	95(60,150)	<0.001	11 (9.3-11.8)	11 (10.05-11.9)	11.5 (9.7-12.5)	0.003
Tranexamic acid + Mefenamic acid (n=3)	590(550,590)	509(300,509)	170(160,170)	0.223	8.6 (7-8.6)	10.5 (9.9-10.5)	10.7 (10-10.7)	0.135
Oral medroxyprogesterone acetate (n=2)	347(280,347)	192(135,192)	70(60,70)	0.135	9.1 (7.7-9.1)	12.2 (11.8-12.25)	12.2 (12-12.25)	0.223
Mefenamic acid (n=2)	244	55	5	0.368	10.3	10.9	11	0.368

**Figure 1:**
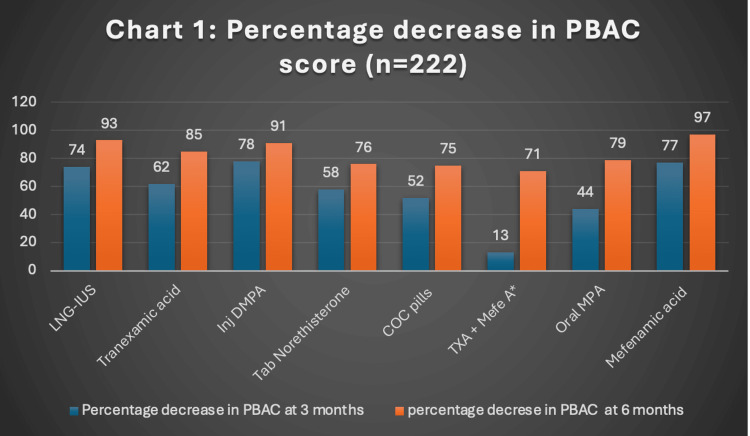
Percentage decrease in PBAC score *Combination of tranexamic acid and mefenamic acid PBAC: Pictorial Blood Loss Assessment Chart; LNG-IUS: levonorgestrel-releasing intrauterine system; DMPA: depot medroxyprogesterone acetate; COC pills: combined oral contraceptive pills; MPA: medroxyprogesterone acetate

The percentage reduction in PBAC scores at three and six months for the various treatment groups was as follows: LNG-IUS (74%, 93%), tranexamic acid (62%, 85%), inj DMPA (78%, 91%), tablet norethisterone (58%, 76%), COCPs (52%, 75%), combination of tranexamic acid and mefenamic acid (13%, 71%), tab MPA (44%, 79%), and mefenamic acid (77%, 97%). The greatest reduction in PBAC score at three months was observed with inj DMPA (78%), while LNG-IUS showed the highest reduction (93%) at six months (Figure 1).

Baseline median hemoglobin (IQR) for various treatment groups was LNG-IUS: 10.2 g/dL (range: 9-11 g/dL), tranexamic acid: 10.6 g/dL (range: 9.3-11.5 g/dL), inj DMPA: 10.3 g/dL (range: 6.8-11.6 g/dL), tab norethisterone: 10 g/dL (range: 8.1-11.8 g/dL), COCP: 11 g/dL (9.3-11.8 g/dL), combination of tranexamic acid and mefenamic acid: 8.6 g/dL (range: 7-8.6 g/dL), and tab MPA: 9.1 g/dL (7.7-9.1 g/dL). A statistically significant improvement in hemoglobin levels (p < 0.001) was observed over six months in the same five treatment groups (Table 3). 

At the end of both three and six months, 217 participants (97.7%) were compliant with the prescribed treatment. Five participants (2.2%) were non-compliant, with reasons including the inconvenience of daily medication intake (OCPs (one); norethisterone (one)), persistent dysmenorrhea despite treatment (norethisterone (one), and early removal of the LNG-IUS by two participants within six months of insertion. 

## Discussion

This study demonstrated a significant reduction in PBAC scores with medical management, indicating a high success rate. Over the course of one year, a total of 18,838 patients attended the gynecology OPD, of which 4,766 (22.3%) were diagnosed with HMB. This aligns with findings from Choudhury et al. [10] and Vaidya et al. [11], who reported HMB prevalence rates of 20.4% and 18.3%, respectively.

The mean age of study participants was 41.03 ± 7.23 years, comparable to the ECLIPSE (Effectiveness and Cost-effectiveness of Levonorgestrel-containing Intrauterine System in Primary care against Standard treatment for menorrhagia) trial conducted by Kai et al. [12], which reported a mean age of 41.9 years. The majority of participants, 101 (42%), were between 41 and 45 years old, while only 3.3% were aged 21-25 years. These findings are consistent with studies by Choudhury et al. [10] and partially with Vaidya et al. [11], who observed the highest prevalence in the 45-49 years group. In a study by Ashworth et al. [13], the highest management rate was among women aged 35-44 years. 

Comorbidities were common, with hypothyroidism present in 28 (11.5%) of participants, followed by diabetes mellitus in 25 (10.3%) and hypertension in 21 (8.6%) participants. In contrast, Vaidya et al. [11] found hypertension to be the most common comorbidity, 68 (30.2%), followed by diabetes and thyroid disorders. Sinha et al. [14] also noted associations with hypothyroidism, heart disease, hypertension, and hepatitis B.

According to the PALM-COEIN classification, 131 (54.5%) of the 240 participants had a structural cause for HMB, with AUB-A being the most frequent, followed by AUB-P. Among non-structural causes, AUB-O was most prevalent. These findings differ from Vaidya et al. [11], who reported a higher proportion of structural causes (77.6%), with leiomyomas (AUB-L) being most common. Similarly, Chaudhary et al. [10] reported leiomyoma as the most common pathology in 26 (26%) cases, followed by AUB-A in 21 (21%) cases.

Regarding treatment modalities, LNG IUS was the most commonly used intervention in 85 (35.4%) participants, followed by tranexamic acid 68 (28.3%), norethisterone 42 (17.5%), DMPA 20 (8.3%), OCPs 18 (7.5%), a combination of tranexamic acid and mefenamic acid 3 (1.2%), and mefenamic acid alone 2 (0.8%). Unlike Raghuwanshi et al. [15], who reported a higher use of ormiloxifene in 121 (40.3%) participants among a total of 300 participants. Our study did not include ormiloxifene as a treatment option. 

Ashworth G et al. [13] reported that in 3264 women with HMB, the COCP was the most frequently prescribed medication (14.7/100 HMB problems), followed by tranexamic acid, norethisterone, and nonsteroidal anti-inflammatory drugs. This differs from the treatment distribution observed in our study. The variation could be attributed to the higher proportion of women aged 35-44 years in their cohort, a group more likely to be prescribed COCPs for the management of heavy menstrual bleeding.

Regarding the efficacy of individual therapies, inj DMPA showed a significant reduction in PBAC score at six months (p<0.001), with a 78% reduction after three months. Hemoglobin improved from 10.3 g/dL to 11.2 g/dL (p= 0.022). Literature suggests DMPA may cause amenorrhea in up to 50% of users after one year [16], though side effects such as weight gain and irregular bleeding can lead to discontinuation [17].

The LNG-IUS showed a 74% reduction in PBAC scores at three months and a 93% reduction at six months (p < 0.05), indicating a significant decrease in menstrual blood loss. Hemoglobin levels also improved markedly. These findings align with those of a similar study by Yu et al. [18], which demonstrated a significant reduction in menstrual volume and an increase in hemoglobin levels in perimenopausal women treated with LNG-IUS for HMB. The difference in the timing of blood-loss reduction between injectable DMPA and the LNG-IUS likely reflects their distinct mechanisms of action. The LNG-IUS delivers a high local concentration of levonorgestrel to the endometrium, leading to initial decidualization and subsequent endometrial atrophy. In contrast, DMPA primarily inhibits follicle-stimulating hormone (FSH) secretion from the anterior pituitary, thereby suppressing ovulation and inducing atrophic changes in the endometrium.

Tranexamic acid achieved a 62% reduction in PBAC score after three months and showed statistically significant hemoglobin improvement at six months (p<0.05). Gupta et al. [19] reported similar outcomes, with a PBAC reduction of 59.6% after three months.

Norethisterone resulted in a 58% reduction in PBAC score at three months and significant hemoglobin improvement at six months (p<0.05). In comparison, Kiseli et al. [20] reported a 47.4% PBAC reduction at three months and 53.1% at six months.

MPA reduced PBAC from 347 to 70 over six months. Hemoglobin increased from 9.1 g/dL to 12.2 g/dL. Kriplani et al. [21] found similar outcomes, with MPA reducing blood loss by 57.7% over three months.

COCPs reduced PBAC score by 52% after three months, with significant hemoglobin improvement at six months (p=0.003). In a randomized controlled trial by Jain et al. [22], a smaller reduction in PBAC score was observed with COCPs, but no significant changes in hemoglobin values were noted. 

For mefenamic acid, among two participants, PBAC scores dropped from 244 to five over six months, with hemoglobin increasing from 10.3 g/dL to 11 g/dL. Reid et al. [23] found LNG-IUS more effective than mefenamic acid, with a larger PBAC score reduction. Eftekhar et al. [24] observed no significant changes in hemoglobin with mefenamic acid.

A major strength of the study is its prospective cohort design, which allowed for systematic data collection over time, enabling evaluation of treatment outcomes at multiple intervals (baseline, three months, and six months). Use of PBAC provided a semi-objective and quantifiable measure of menstrual blood loss. Inclusion of various commonly used medical therapies allowed comparative evaluation of effectiveness and compliance. In addition to clinical outcomes, the study also investigated patient compliance and reasons for nonadherence, offering a more holistic understanding of treatment effectiveness.

Limitations

The study was conducted at a single tertiary care institution, which may limit the generalizability of the findings to other settings. A follow-up duration of six months may not fully capture long-term efficacy, side effects, or continued compliance. Patients self-selected their treatment modality, which may introduce selection bias and affect comparability between groups. Finally, the PBAC score used to estimate menstrual blood loss is a semi-objective tool that depends on the patient’s subjective interpretation of pad staining. 

Scope for further research 

Future studies focusing on long-term follow-up to assess the effectiveness, side effects, and continued compliance of various medical therapies for HMB should be conducted. Also, comparative studies involving larger and multicenter populations, stratification by age and BMI, and randomized controlled trials would strengthen the evidence. 

## Conclusions

The study confirms that overall medical management was highly effective, particularly with LNG-IUS, tranexamic acid, and inj DMPA in reducing menstrual blood loss and improving hemoglobin levels in patients with HMB. Among these, LNG-IUS demonstrated the greatest reduction in PBAC scores, indicating superior efficacy, though the cost and availability may limit its broader use. Compliance rates were highest with LNG-IUS, likely due to its long-acting nature and minimal need for daily intervention, whereas oral medications like tranexamic acid showed relatively lower adherence. The choice of therapy should be individualized, taking into account patient characteristics, comorbidities, treatment response, and likelihood of compliance. 

## Figures and Tables

**Figure 2:**
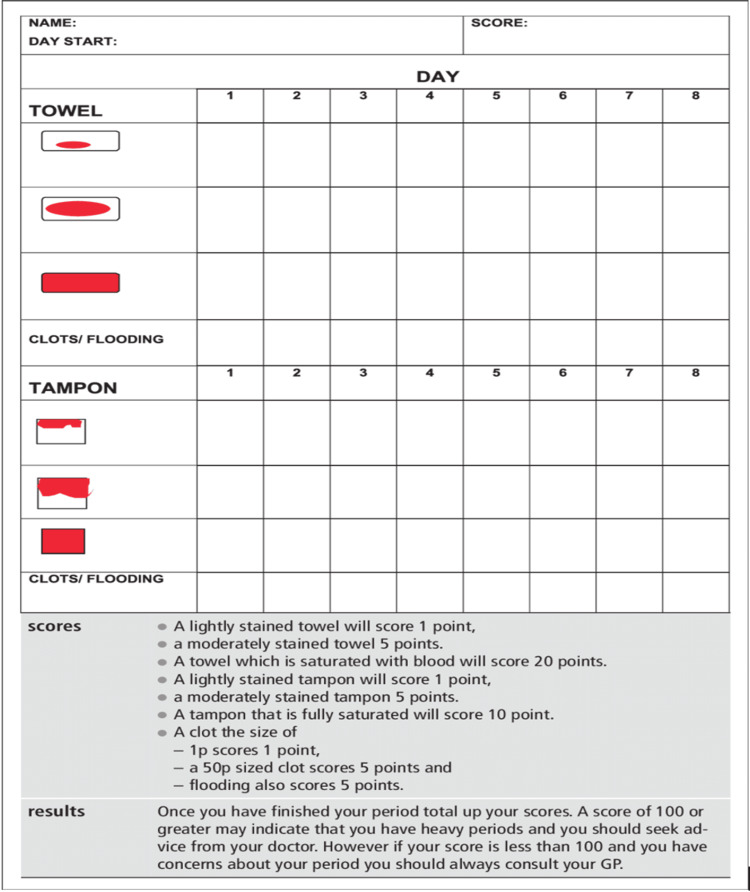
Pictorial Blood Loss Assessment chart and scoring system for assessment of menstrual blood loss Reproduced with permission from the authors: Higham JM, O’Brien PMS, Shaw RW. Assessment of menstrual blood loss using a pictorial chart. BJOG: An International Journal of Obstetrics & Gynaecology. 1990;97:734–739. [9]

## Appendices

Appendix A

This is the semi-qualitative method of assessment of blood loss. The chart is scored using a scoring system devised by Hingam et al. [9]. Diagrams of clot sizes were modified to sizes of local coins (5 cent - Rs 1 coin and 50 cent - Rs 2 coin). Participants were instructed to note the number of pads/tampons/towels used per day and the degree of soiling of the product. The participant had to mark accordingly as given in the chart (Figure 2). 
